# Fatal Oropouche Virus Infections in Nonendemic Region, Brazil, 2024 

**DOI:** 10.3201/eid3011.241132

**Published:** 2024-11

**Authors:** Antonio Carlos Bandeira, Felicidade Mota Pereira, Arabela Leal, Sara P.O. Santos, Ana Claudia Barbosa, Marcia Sao Pedro Leal Souza, Daniele Ribeiro de Souza, Natalia Guimaraes, Vagner Fonseca, Marta Giovanetti, Luiz Carlos Junior Alcantara, André Alvarez A. Lessa, Ramon Costa Saavedra, Luiz Marcelo R. Tomé, Felipe Campos M. Iani, Rivia Mary Barros, Sandra Maria O. Purificação, Jaciara Prado de Jesus, Ricardo Rosário Fonseca, Marcio Luis Valença Araújo

**Affiliations:** Laboratório Central de Saúde Pública da Bahia, Salvador, Brazil (A.C. Bandeira, F. Mota Pereira, A. Leal, S.P.O. Santos); Diretoria de Vigilância Epidemiológica do Estado da Bahia, Salvador (A.C. Barbosa, M.S.P.L. Souza, D.R. de Souza, A.A.A. Lessa, R.C. Saavedra, S.M. de Oliveira da Purificação, J.P. de Jesus, M.L.V. Araújo); Fundação Ezequiel Dias, Belo Horizonte, Brazil (N. Guimaraes, F.C.M. Iani); University of the State of Bahia, Salvador (V. Fonseca); Universita Campus Bio-Medico di Roma, Rome, Italy (M. Giovanetti); Oswaldo Cruz Institute, Oswaldo Cruz Foundation, Rio de Janeiro, Brazil (M. Giovanetti); Instituto Rene Rachou, Fundação Oswaldo Cruz, Minas Gerais, Brazil (L.C.J. Alcantara, L.M.R. Tomé); Superintendência de Vigilância em Saúde, Salvador (R.M. Barros); Santa Casa de Misericórdia de Valença Hospital, Valença, Brazil (R.R. Fonseca); Instituto Federal de Educação, Salvador (M.L.V. Araújo)

**Keywords:** Oropouche virus, viruses, vector-borne infections, Oropouche fever, outbreak, Bahia, Brazil

## Abstract

We report acute Oropouche virus infections in 2 previously healthy women from a nonendemic region of Brazil outside the Amazon Basin. Infections rapidly progressed to hemorrhagic manifestations and fatal outcomes in 4–5 days. These cases highlight the critical need for enhanced surveillance to clarify epidemiology of this neglected disease.

Oropouche virus (OROV), the etiologic agent of Oropouche fever, is an arbovirus that belongs to the *Orthobunyavirus* genus of the Peribunyaviridae family (*1*). Discovered in 1955 in Trinidad and Tobago, the virus subsequently was isolated from a pale-throated sloth (*Bradypus tridactylus*) in Brazil in 1960 (*2*,*3*). Transmission to humans in urban settings is thought to occur mainly through the bites of infected *Culicoides paraensis* midges (*4*).

In 2020, a few OROV cases were retrospectively detected in the Salvador metropolitan region, Bahia state, Brazil (*5*), and OROV was considered nonendemic that region. However, in March 2024, the Central Public Health Laboratory detected OROV in Bahia again (*6*). Since then, a major outbreak has erupted in parallel with increasing case numbers in Brazil (*6*), but severe outcomes have not been reported. We report 2 cases of Oropouche fever in Bahia that progressed to death.

## The Study

We retrospectively collected clinical information by analyzing digital records and conducting an epidemiologic investigation to collect clinical and laboratory data. In addition, we conducted interviews with the medical teams who cared for the patients and investigated residents living in the same households as the case-patients. The study was approved by the Brazil National Research Ethics Commission (approval no. CAAE 81053724.6.0000.0052).

Patient 1 was a 24-year-old woman whose symptoms began with fever lasting 1 day, headache, retroorbital pain, myalgia, severe abdominal pain, diarrhea, nausea, and vomiting. She had no underlying conditions, was not pregnant, and had history of miscarriage, and was admitted 3 days after symptom onset due to worsening symptoms and blurred vision. She continued to report severe abdominal pain and hypoactivity and had ocular edema 7 hours after admission.

At 10 hours after admission, psychomotor agitation developed, and in the subsequent 2 hours the patient began to experience hypotension and desaturation. Clinicians introduced a Venturi mask at 8 liters of oxygen per minute, followed by orotracheal intubation, when bronchial hemorrhage was detected. One hour later, the patient progressed to cardiorespiratory arrest and died the next day, 13 hours after admission. Samples collected at 6 hours and 13 hours after admission (4 days after symptom onset) showed rapid decline in hematocrit, thrombocytopenia, and prolongation of clotting time, as well as elevated liver enzymes and renal dysfunction (Table 1).

**Table 1:** Laboratory results for patient 1 after admission in a case of fatal Oropouche virus infection in nonendemic region, Brazil, 2024*

Variable	Time after admission	
	6 hours	13 hours
Hematocrit, %	50.3	20.9
Hemoglobin, %	16.7	7.0
Mean corpuscular volume, fL (reference <80 fL)	82	88
Mean corpuscular hemoglobin, pg (reference 27–31 pg)	27	29
Leukocytes, cells/mm^3^	44,700	24,500
Neutrophils, %	71	80
Band forms, %	8	10
Metamyelocytes, %	1	1
Lymphocytes, %	16	6
Platelets, cells/mm^3^	125,000	43,000
Bleeding time, min	ND	1
Clotting time, min	ND	>30
Clot retraction	ND	Complete
Aspartate aminotransferase, U/L	ND	970
Alanine aminotransferase, U/L	7	404
GGT, U/L	559	144
TB/DB, mg/dL	2.78/1.52	ND
Creatinine, mg/dL	4.1	2.3

*GGT, gamma-glutamyl transferase; ND, not done; TB/DB, total bilirubin/direct bilirubin.

Patient 2, a 21-year-old woman, had fever, myalgia, headache, retroorbital pain, pain in the lower limbs, asthenia, and joint pain. After 4 days, a rash and purple spots on her body developed, as did nose, gum, and vaginal bleeding. The patient reported weakness, drowsiness, and vomiting. She had no underlying conditions, denied pregnancy or previous miscarriage, and was admitted to a local hospital. After 9 hours she was transferred to a secondary facility and appeared drowsy, had cyanosis of the extremities and persistent vomiting, and had not eaten in several days. On examination, she had bleeding gums and epistaxis, vaginal bleeding, and cold and clammy skin, in addition to widespread petechia. She died 2 hours later. Samples collected 5 days after symptom onset showed thrombocytopenia, prolongation of clotting and bleeding time, and renal dysfunction (Table 2). A household member retrospectively had Oropouche fever confirmed.

**Table 2:** Laboratory test results for patient 2 in a case of fatal Oropouche virus infections in nonendemic region, Brazil, 2024*

Variable	Time after admission	
	At admission	10 hours
Hematocrit, %	38.7	43.7
Hemoglobin, %	13.5	14.0
Mean corpuscular volume, fL (reference <80 fL)	86	82
Mean corpuscular hemoglobin, pg (reference 27–31 pg)	30	26
Leukocytes, cells/mm^3^	9,500	19,400
Neutrophils, %	59	58
Band forms, %	0	0
Metamyelocytes, %	0	0
Lymphocytes, %	34	36
Platelets, cells/mm^3^	147,000	91,000
Prothrombin time, sec	ND	>120
Partial thromboplastin time, sec	ND	>120
Bleeding time, min	ND	5
Clotting time, min	ND	>30
TB/DB, mg/dL	ND	2.71/1.54
Creatinine, mg/dL	ND	3.6
NS1	Nonreactive	ND

*Patient was transferred to a second hospital after initial admission. ND, not done; NS1, rapid immunochromatographic test for dengue virus; TB/DB, total/direct bilirubin.

We used Extracta Kit DNA and RNA of Pathogens (Loccus, https://www.loccus.com.br) to extract genetic material from 200 μL of clinical samples, following manufacturer’s instructions. Subsequently, we conducted real-time reverse transcription PCR (RT-PCR) reactions for different pathogens. We used inputs produced by the Institute of Molecular Biology of Paraná (IBMP) for quantitative RT-PCR (qRT-PCR) for OROV, as previously described (*7*).

To differentiate Oropouche diagnoses, we conducted RT-PCR for other pathogens. For Mayaro virus, we used RT-PCR techniques from IBMP (*7*). For *Leptospira*, we used an in-house RT-PCR method for detecting the lipL32 target gene. We used the ZC D-Typing Molecular Kit (Bio-Manguinhos, https://www.bio.fiocruz.br) for Zika, chikungunya, and dengue viruses. For *Hemophilus influenzae*, *Neisseria meningitidis*, and *Streptococcus pneumoniae*, we used the Viasure PCR Detection Kit (Certest Biotec, https://www.certest.es) (Table 3).

**Table 3:** Molecular biology and serologic test results in 2 cases of fatal Oropouche virus infection in nonendemic region, Brazil, 2024*

Laboratory test	Patient 1	Patient 2
qRT-PCR		
Dengue virus	Undetectable	Undetectable
Chikungunya virus	Undetectable	Undetectable
Zika virus	Undetectable	Undetectable
Mayaro virus	Undetectable	Undetectable
Oropouche virus, Ct value	Detectable, 16	Detectable, 8
qPCR		
* Leptospira*	Not done	Undetectable
* Neisseria meningitidis*	Undetectable	Undetectable
* Streptococcus pneumoniae*	Undetectable	Undetectable
* Hemophilus influenzae*	Undetectable	Undetectable
Serology		
Dengue virus IgM	Nonreactive	Nonreactive
*Leptospira* IgM	Nonreactive	Nonreactive
Chikungunya virus IgM	Nonreactive	Nonreactive
Hepatitis C virus IgG, IgM	Not done	Nonreactive
Hepatitis B virus IgG, IgM	Not done	Nonreactive
Hepatitis A virus IgG, IgM	Not done	Nonreactive


*Performed in serum samples, except for *Leptospira* qPCR, which was performed on whole blood. Patient 1 samples collected 4 days after symptom onset; patient 2 samples collected 5 days after symptom onset. Ct, cycle threshold; qPCR, quantitative PCR; qRT-PCR, quantitative reverse transcription PCR.

For serologic tests (Table 3), we used Panbio Dengue IgM Capture ELISA (Abbott Point of Care, https://www.globalpointofcare.abbott) for dengue virus (DENV); Anti-Chikungunya virus ELISA (IgM) (EUROIMMUN, https://www.euroimmun.com) for chikungunya; and Panbio Leptospira IgM (Abbott Point of Care) for *Leptospira*. For hepatitis viruses, we used serologic tests from Roche Diagnostics (https://diagnostics.roche.com), including Elecsys HBsAg II and Elecsys Total Anti-HBc II for hepatitis B Elecsys Anti-HCV II for hepatitis C, and Elecsys Anti-HAV for hepatitis A and IgM. We used all kits in accordance with the manufacturers’ guidelines.

We sequenced samples using the viral metagenomics approach, according to the SMART-9N protocol (*8*). Initially, we subjected samples to nucleic acid extraction for DNA and RNA and concentrated to 10 µL by using Zymo RNA Clean and Concentrator-5 (Zymo Research, https://www.zymoresearch.com). Next, we performed cDNA synthesis by using SuperScript IV Reverse Transcriptase (Thermo Fisher Scientific, https://www.thermofisher.com) and random primers RLB RT 9N and RLB TSO synthesized in-house (I.C. Morales, unpub. data, https://doi.org/10.17504/protocols.io.7w5hpg6). We prepared the sequencing library by using the Ligation Sequencing Kit (SQK-LSK109) and Native Barcoding Kit (Oxford Nanopore Technologies [ONT], https://nanoporetech.com). We loaded the final 60-ng library onto an R9.4.1 flow cell (ONT) and sequenced for 24 hours on the MinION nanopore sequencer (ONT). We used the Genome Detective pipeline (https://www.genomedetective.com) to assemble raw reads. We aligned all sequences by using MAFFT (*9*) and manually edited by using AliView (*10*). To explore the relationship between the sequenced genomes obtained in this study and those sampled globally, we constructed maximum-likelihood phylogenies for the small, medium, and large segments by using IQ-TREE 2 software under the Hasegawa-Kishino-Yano plus gamma 4 substitution model (*11*).

Sequencing the complete genome enabled generation of complete genomes of 3 segments. Analysis revealed that the genomes clustered with sequences recently isolated from the northern part of Brazil (F. Naveca et al., unpub. data, https://doi.org/10.1101/2024.07.23.24310415) (Figure). We did not identify any novel mutations. However, we plan further comparisons during this ongoing outbreak to check for point mutations.

**Figure:**
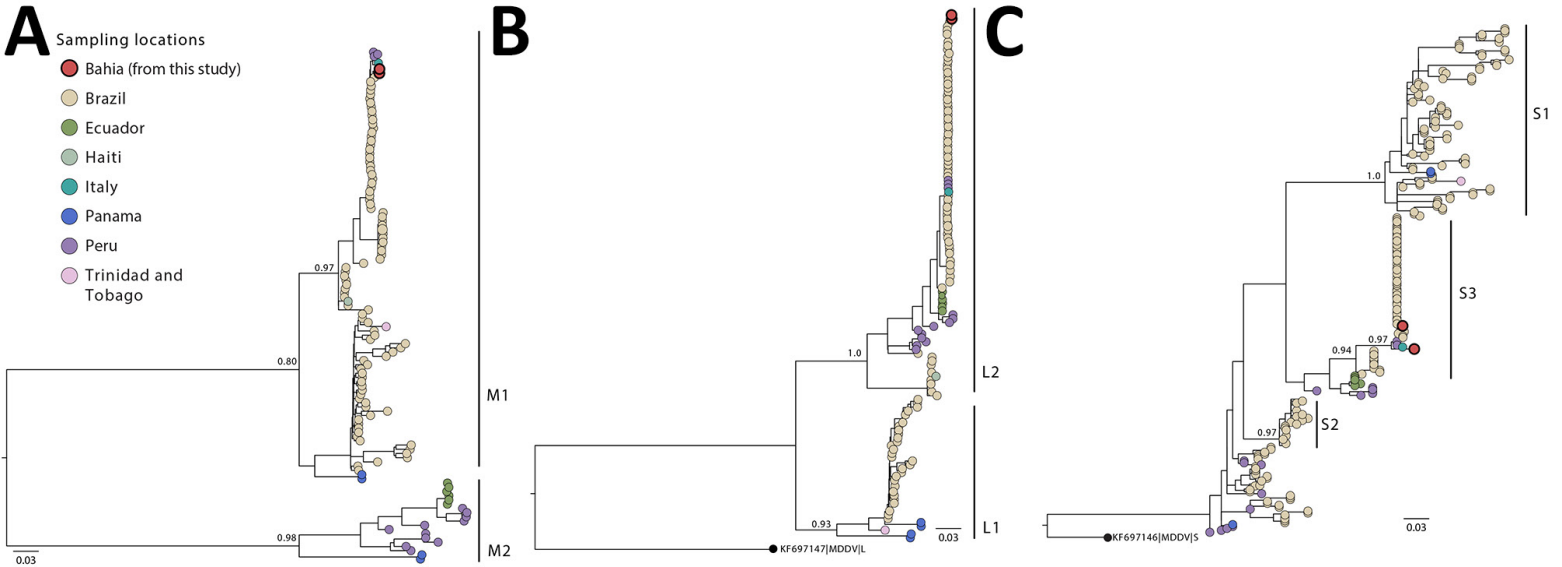
Maximum-likelihood phylogenetic trees of the 3 independent OROV segments from fatal Oropouche virus infections in nonendemic region, Brazil, 2024. A) Medium segment (n = 122); B) large segment (n = 138); C) small segment (n = 264). Tips of prototypical viruses and major clusters are color-coded according to locations of isolation. The trees included annotations indicating the bootstrap probability support for both major lineages and specific clades. Trees were constructed by using IQ-TREE 2 software under the Hasegawa-Kishino-Yano plus gamma 4 substitution model (*11*). MDDV was included as an outgroup in the large and small segment trees. Scale bars indicate nucleotide substitutions per site. MDDV, Madre de Dios virus; OROV, Oropouche virus.

## Conclusions

By March 2024, an OROV outbreak was spreading in Bolivia, Colombia, Peru, and Cuba, and >7,800 cases were detected in Brazil (*12*). However, the clinical course of the 2 cases we describe highlights the possibility for rapid evolution from symptom onset to death in 4­–5 days. In addition, severe coagulopathy was the probable mechanism that led to death, and we observed evidence of liver and kidney involvement that may have contributed to the coagulopathy and, consequently, to death.

One previous study observed hemorrhagic phenomena in 20 patients (15.5% of the sample) but did not present laboratory data (*13*). Another study demonstrated that OROV could be detected in the liver 6 hours after OROV was intracerebrally inoculated into 3-week-old hamsters (*14*), suggesting hematogenous virus transmission from the brain to liver lesions and substantial hepatocyte necrosis.

In both cases we describe, the clinical course was remarkably like that of severe dengue, but the mechanisms that triggered the events leading to death remain unknown. Our 2 case-patients did not share any family or household links, lived in different cities, and did not have any underlying conditions that would increase their risks for severe disease. Furthermore, coinfection with DENV is unlikely because the RT-PCR we used has a 97.3%–100% specificity for DENV, and having 2 undetected dengue cases by that assay is unlikely. Finally, we sequenced the samples using viral metagenomics and only identified OROV.

In conclusion, we describe clinical and laboratory findings and phylogeny from 2 fatal cases of OROV infection in the nonendemic region of Bahia, Brazil. An OROV outbreak continues to expand in the Americas, and our findings underscore the urgent need to clarify the pathophysiology of this neglected disease.
